# Empowerment in outpatient care for patients with chronic kidney disease - from the family member's perspective

**DOI:** 10.1186/1472-6955-10-21

**Published:** 2011-10-28

**Authors:** Annette Nygårdh, Kerstin Wikby, Dan Malm, Gerd Ahlstrom

**Affiliations:** 1 The Department of Nursing, School of Health Sciences, Jönköping University, Jönköping, Sweden; 2 The School of Health and Caring Sciences, Linneus University, Växjö, Sweden; 3 The Department of Internal medicine, County Hospital Ryhov, Jönköping, Sweden; 4 The Swedish Institute for Health Sciences, Lund University, Lund, Sweden

## Abstract

**Background:**

Family members of persons with pre-dialysis chronic kidney disease may experience feelings of vulnerability and insecurity as the disease follows its course. Against this background, the aim of the present study was to explore empowerment in outpatient care as experienced by these family members.

**Methods:**

An inductive approach for qualitative data analysis was chosen. The study sample comprised 12 family members of pre-dialysis patients at an outpatient kidney clinic. Two interviews with each family member were subjected to content analysis to gain an understanding of empowerment from the family members' perspective.

**Results:**

*Having strength to assume the responsibility *was the main theme that emerged from the following five sub-themes: Being an involved participant, Having confirming encounters, Trusting in health-care staff, Comprehending through knowledge, and Feeling left out. Four of these five sub-themes were positive. The fifth subtheme illuminated negative experience, indicating the absence of empowerment.

**Conclusions:**

Family members' experience of empowerment is dependent on their ability to assume the responsibility for a relative with chronic kidney disease when needed. The findings emphasise the need for a family perspective and the significance of a supportive environment for family members of persons in outpatient care.

## Background

Research on families of adults with chronic diseases has increased over the last decade, perhaps related to recognition of the family's importance in providing psychological support for the patient and fulfilling the role of caregiver [1]. However, studies specifically about the families of patients with chronic kidney disease are sparse [2]. More general research regarding family members living with a person with a chronic disease reveals a reduced sense of individual freedom arising from the responsibility for the care of the patient [3-5]. Furthermore, it is common for family members to put their own needs in the background, and they describe a vulnerability involving a sense of stigmatization, loss and anxiety [3].

Research has shown that family members of persons with serious chronic illness in outpatient care view their future as insecure and frightening. They appreciate information that helps them understand disease-related changes in the patient [5]. Family members avoid burdening others with their problems because they think they would not understand anyway. They feel ashamed of their feelings of fatigue, anger, and frustration and are afraid of being perceived by others as inadequate or bad partners. Staying in control and handling the situation within the family is important to family members. However, sometimes they feel lonely, and they admit that gaining strength from the support of people outside the family is important. They express a need for closeness to significant others to counteract feelings of insecurity [5].

Important for the family members' sense of control is personal contact with health-care professionals, adequate information and continuity. Moreover, being respected and listened to play a decisive role in the family members' confidence [4]. Lack of insight into the disease and lack of support from professionals make it difficult to live with a person with a chronic disease [6]. Furthermore, according to previous research, it is important that family members get the same information as the patient to facilitate their understanding of the person's situation [7].

Family members of patients undergoing dialysis are struggling to maintain control over their daily lives and have a constant concern about their family member's condition. Previous research has shown that family members experience a fragmented existence by constantly feeling they must be available to support the patient's medical situation and dialysis treatments. Consequently, family members are less free to plan their own activities [8]. They neglect their own health to give priority to the patient's needs [9]. They describe a sense of uncertainty about the future and lack of knowledge regarding treatment available for the patient [10]. Moreover, family members describe feelings of frustration and powerlessness associated with their awareness of the disease prognosis [8].

Empowerment in nursing implies an emphasis on mutual participation, knowledge acquisition, equal partnership [11] and mutual decision-making regarding health issues and goals [12]. Gibson (1991) describes empowerment as a process that includes both separate and interconnected patterns of behaviour of the individuals involved. It implies mobilization and enhancement of individuals' own resources to enable them to feel in control of their lives, able to meet their own needs and solve their own problems [13].

Family members of persons with pre-dialysis chronic kidney disease in out-patient care feel vulnerable and insecure as the disease follows its course [14,15]. They have to manage the consequences of the patient's decline in bodily functions, loss of energy and diet restrictions resulting from the disease [14,16,17]. Moreover, they have to cope with the patient's emotional feelings, low acceptance of the disease and loss of personal control over the illness [17,18]. Family members' experiences of empowerment in relation to outpatient care have not been described in the literature up to now. Interviewing family members about such experiences offers a means of providing health-care professionals with a better basis for offering these people adequate support in their endeavour to achieve empowerment. In line with this, the aim of the present study was to explore empowerment in outpatient care as experienced by family members of persons with pre-dialysis chronic kidney disease.

## Methods

### Design

An inductive qualitative interview [19] study with latent content analysis [20,21] was chosen to illuminate family members' experiences of empowerment in the context of chronic kidney disease outpatient care. This design was chosen because of its appropriateness for capturing the phenomena from the individual's perspective [22].

### Sample and participants

Family members who participated in this study were Swedish and purposefully selected by patients with pre-dialysis chronic kidney disease who had participated in an earlier study [23]. The patient made known to the first author (AN) the name, address and phone numbers of the family member chosen as a possible participant. The first author received information for a total of 20 family members, and all were asked to participate in the study. Eight of them declined because of lack of insight into health care, lack of time or lack of interest. Thus, 12 family members participated: 8 women and 4 men 32-67 years old (median age 61). Further background data are provided in Table 1. Two participants were not interviewed a second time, one of them (wife, age 56) because she did not have the time and the other (daughter, age 63) because she was unreachable.

**Table 1:** Background data of the participants

Background	Interview 1 (n = 12)	Interview 2 (n = 10)
*Relationship to CKD patient*		
Husband/Wife	7	6
Son/Daughter	3	2
Father/Mother	1	1
Brother/Sister	1	1
		
*Gender*		
Male	4	4
Female	8	6
		
*Education*		
Compulsory school	3	2
Upper secondary school	4	4
University	5	4
		
*Employment status*		
Employed	9	7
Retired	2	2
Student	1	1
		
*Place of Residence*		
Urban	5	5
Rural	7	5

### Procedure and interviewing

A letter was sent to the selected family members explaining the purpose of the study and the meaning of informed consent and confidentiality. Within two weeks from receipt of the letter the family members received a telephone call giving further explanation and offering the opportunity to ask any questions they might have prior to their decision about participation in the study. The data were collected from April 2009-May 2011, on two occasions for each participant, with approximately two years between the interviews. The places for the interviews were chosen by the participant to ensure the most convenient environment. Interviewers attempted to create a dialogue designed to capture rich narrations of the family members' experiences. In the case of the first interview, performed by the second author (KW), the participants were interviewed at their home (n = 7), at the hospital (n = 1), in a public place (n = 1) or at their workplace (n = 3). The interview was carried out in the form of a dialogue [24] that lasted 20-45 minutes and started with the following open-ended question: "Could you tell me, please, about your experiences in relation to your sick relative's health care?" The number and formulation of follow-up questions depended on the richness of the participant's answer to the open question. The follow-up questions emanated from three areas: experiences of involvement, experiences of self-determination and awareness of the care process.

The procedure with regard to the second interview, performed by the first author (AN), included listening to the first interview and making notes on content areas [21], this in an effort to obtain more comprehensive data concerning these areas. This preparatory step was performed directly before the second interview. The interview lasted 35-90 minutes and was carried out as mentioned above, starting with the same open-ended question as in the first interview. The participants were interviewed at their home (n = 6), at their workplace (n = 3), or in the first author's office (n = 1). All interviews were digitally recorded and then transcribed verbatim.

### Data analysis

Qualitative inductive content analysis [20,21] was chosen to gain an understanding of empowerment from the family members' perspective. The analysis of all the transcribed interviews was performed in several steps. The first step was an "open" reading of each interview to obtain an overall impression of its content. In the second step, meaning units with reference to the participant's experiences of health care were identified from the transcribed data. A meaning unit consisted of one or more sentences or paragraphs of a narrative. In the third step, the meaning units were condensed (keeping close to the text), and in the fourth step, the interpretation of the underlying meaning was expressed in terms of codes [25]. The co-authors read the first author's initial analysis. The condensations and codes were then subjected to critical discussion within the research group, resulting in certain modifications of the codes. Thereafter, in the fifth step, the codes were analysed and labelled by the first author (AN) into sub-themes. After reading the analysis as a whole, all the authors discussed and compared the findings until agreement was reached. The interviews in their entirety served as a point of reference throughout the analytical process when deeper understanding was needed of the meaning units, codes and sub-themes. In the sixth and last step, one theme was developed from the sub-themes, expressing the main thread or main latent content of the text [21].

### Ethics

The study was approved by the Research Ethics Committee at Linkoping University in Sweden (*Dnr: M205-08*). The participants were informed that they could withdraw their participation at any time without providing any explanation and without any consequences for future care of the patient or themselves. Confidentiality was guaranteed through coding of the transcripts of the interviews and presentation of the data at group level. The participants provided written informed consent before the interviews.

## Results

The participants' descriptions resulted in one theme: *Having strength to assume the responsibility *and five sub-themes (Table 2), which together are the contents of what empowerment is in outpatient care, as experienced by family members of persons with pre-dialysis chronic kidney disease. Four of these five sub-themes are positive: *Being an involved participant, Having confirming encounters, Trusting in health-care staff *and *Comprehending through knowledge*. The fifth, *Feeling left out*, is negative and has to do with the absence of empowerment.

**Table 2:** Empowerment experienced by family members of persons with pre-dialysis chronic kidney disease in outpatient care

Theme	Sub-themes
	Being an involved participant
	Having confirming encounters
Having strength to assume the responsibility	Trusting in health-care staff
	Comprehending through knowledge
	Feeling left out

### Having strength to assume the responsibility

Having strength to assume the responsibility was supported by the family members' experiences of being an involved participant in the patient's care. Experiences of being involved represented the ability to share the patient's experiences and follow the course of patient care. Furthermore, empowerment was supported when there were confirming encounters with health care staff. It was essential to their experience of empowerment to be treated with respect and taken into account in the patient's care. The family members expressed satisfaction with the care received and felt they could trust the staff and their ability. Family members reported that it was essential that staff approach the patient by demonstrating respect for the resources brought as an individual to the care situation and not "talk over the patient's head". Family members also emphasised the importance of health-care staff recognizing their need to comprehend. Specific individual information given by the staff helps family members understand the impact of the disease on the patient and puts them in a better position to support the person. There was also reference to empowerment in terms of its *absence*: family members sometimes felt they were being left out, this having to do with their lack of knowledge concerning how to support the patient and their sense of not being a natural partner in health care.

#### 1. Being an involved participant

Being an involved participant in the patient's care meant having respect for the person's autonomy and protecting their relative from anxiety. Family members described experiences of being an equal partner in dialogues regarding emotional issues connected to the illness and in health-care decision-making. One aspect of being an involved participant was feeling connected to others in similar situations. The family members wanted to share the patient's care experience and be afforded the opportunity to follow the care process. They expressed a willingness to act and assume responsibility when necessary. They could, for example, manage the person's medication. Being a positive force concerning change of lifestyle habits was important for the empowerment of the family members as they felt that it had a positive impact on the patient's condition.

*"I took it up with the doctor again a bit today, so he tried to get round to talking about exercise [with my husband]. I mean, I think his whole body's going downhill. You just feel it -- I can see just where it's going if he doesn't take care of himself. That's the really hard part, living with someone who doesn't think about this all the time." *(wife, age 67)

#### 2. Having confirming encounters

Having access to health-care staff and their help was important for the family members' experiences of confirmatory encounters. It was essential that there was personal contact with staff where family members were recognized and addressed by name. Being respected as a person with a valuable part to play in the patient's care gave a sense of confirmation as did feeling that one's worries concerning the consequences of the disease were being taken seriously. The family members spoke of the importance of sharing their knowledge with staff. A significant aspect of confirming encounters was the nursing staff's positive attitude and commitment, in terms both of kindness and willingness to answer questions. Family members very much appreciated a quick response to their questions and concerns.

*"Right from the start they made it clear that if there's anything we're wondering about or worrying about, we can just call them up and ask. They're friendly when you ring; you don't feel you're disturbing them or anything. The same way as they do their very best to find out all they can so that you can get the answer you're looking for." *(husband, age 64)

#### 3. Trusting in health-care staff

Trusting in health-care staff was important to the family members' sense of empowerment. It was predicated on the belief that the patient was receiving adequate care. It was also related to the family members' earlier experiences of being treated with empathy by the staff. The experience of continuity in health care created a feeling of security and trust, as did the experience of co-ordination of treatment and needs. Trust in the staff's competence was indispensable. The family members derived a sense of security from the staff's assuming the utmost responsibility in decisions regarding the patient's care. Honest information about the person's medical condition was essential to their trust in the staff. Furthermore, there was trust in the staff even though the family members sometimes had experiences of failure concerning the care.

*"Of course they've got an enormous amount of knowledge and so they can do an awful lot, but of course not everything. Things can go wrong, but generally speaking I've got great trust in them." *(son, age 48)

#### 4. Comprehending through knowledge

Comprehending through knowledge was important for the family members' experiences of empowerment. They spoke of the importance of getting answers to their questions to increase their knowledge about the disease. It was also important that they had sufficient knowledge to understand the patient's mental and physical reactions attributable to the disease. They sought knowledge via the Internet, by talking to the patient and the health-care staff or by reading the literature. The latter could concern not only the disease, but also such things as preventive activities or diet. Through the literature they discovered more about such concerns as the effect of the disease on the cognitive capacity of the patient. Comprehending through knowledge was promoted when they received information that was tailored to the course of the patient's disease. Uncertain about the future, they wanted to acquire knowledge about the consequences of the disease to be better prepared. Comprehension through knowledge about the disease was also created by the family members' being afforded the opportunity to participate in medical visits and thereby get answers to their questions.

*"I usually sent along a few questions for him to ask, otherwise he just asks the usual ones he always asks, about levels and that sort of thing. I perhaps ask a bit more about how it's developing and whether there's anything I should look out for, and whether what he eats is important, and what we can expect. He doesn't ask things like that." *(wife, age 60)

#### 5. Feeling left out

The family members also spoke about the absence of empowerment, in terms of their having a sense of feeling left out. They felt that there were limitations in access to health care and that the staff decided the time for the meeting, which was difficult to change. When family members did not have the opportunity to participate in health-care visits they were less able to share the patient's experiences. Furthermore, family members spoke of being unable to obtain the same information as the patient. They experienced a limited ability to influence the treatment and felt that they were considered unauthorized and not listened to. Feeling left out meant not getting health-care staff's support with regard to being prepared for the difficult decisions, for example, organ donation. Limited in their knowledge about the disease, they were limited in their ability to support the patient. They also experienced uncertainty about their role in the care. Family members described experiences of feeling left out because of the nature of the most common structures and processes of care.

*"When it comes to the whole apparatus of care, well, it is what it is. There's not really anything to think about, the limitation's just part of the system. You get socialized into it." *(wife, age 32)

Finally, when regarding the content of all interview texts, there were observed differences between the participants' narrations. Parents and children of the patient most strongly emphasised trust in health-care staff and the urgency of the patient's need for empowerment to take control of the management of the disease as long as possible. However, they were willing to be supportive when the patient asked for help or complained about care received.

## Discussion

The overall theme that emerged in this study, *Having strength to assume the responsibility*, characterizes what the family members describe as empowerment. Each sub-theme was a distinct part of the more comprehensive theme, but in the same way overlapping in terms of narrations about the family members, patients, and health-care resources and abilities. Family members spoke of the importance of understanding the patient's physical and emotional condition as the disease progressed. They achieved empowerment when they felt able to support the patient. It was important to take into account the person's ability by showing respect and awaiting their expressed need for help. Empowerment also comes from confidence in the health-care staff to provide high-quality care. A literature review [26] concerning family members' empowerment in palliative care shows that partnership and involvement in decision-making about care is regarded as empowerment. Wahlin and colleagues (2009) found in the context of intensive care that involvement in the care process was an important aspect of family members' empowerment [27]. To the best of our knowledge, however, the present study reports the first findings related to empowerment in outpatient care as experienced by family members of persons with pre-dialysis chronic kidney disease.

It was found that confirming encounters with health-care staff were important for the family members' empowerment. There were accounts of being treated with respect and being regarded as a valued person in respect to the  patient's care. Research on the close family of patients in inpatient care has shown the importance of being met with respect and being listened to, both from the point of view of the family members' sense of control [4,27] and quality of care [28]. Furthermore, empowerment in nursing has been defined in terms of respect, equal partnership and mutual participation [11-13]. Findings in this study emphasise the health-care staff's role in increasing family members' sense of empowerment through confirmatory encounters. Furthermore, the findings support previous research [5] showing that family members express the need to be met with understanding and receive emotional support.

Trust in the health-care staff was found to be of importance for family members' experiences of empowerment. Research regarding intensive care has shown that a caring atmosphere where family members receive continuous and honest information increases their empowerment, as does the belief that the patient is receiving the best medical treatment possible [27]. Millberg and colleagues [29] found that family members felt powerless when the patient's diagnosis was delayed and there was difficulty getting help.

The family members in this study described knowledge acquisition as essential for their empowerment, a finding supported by previous research [4]. Comprehending through knowledge was accomplished by getting answers to their questions. They sought information from the literature, via the Internet, and by talking to health-care staff. Seeking information outside health-care encounters to acquire an understanding of the disease has been described by patients before [23] and is in accordance with the process of establishing empowerment. Previous findings have underlined the need for staff to support family members by providing them with relevant knowledge that is important to them [6,13,30].

Empowerment can also be highlighted through consideration of its opposite: powerlessness [31]. The family members in this study described experiences of feeling left out. They experienced limitations with regard to being invited to attend the patient's health-care encounters, and they were uncertain about their role in the person's care, this because of health-care structures and processes. Previous research in critical care has illuminated the importance of the family members' feeling of participation, enabling them to gain control over the situation [32], ease their burden [5], decrease their vulnerability [33] and give them strength [4]. The inability to obtain the same information as the patient, referred to by family members in the present study, represents the absence of empowerment [11-13]. Previous research supports this finding of powerlessness in terms of being unable to help the patient and having no other choice than to rely on the health-care professionals [32] even if they are not easily accessible.

The present findings regarding empowerment for family members include the importance of having the knowledge and ability to be supportive as well as having the willingness to act and assume responsibility when necessary. These findings emphasise the need for a health-care organization that includes the family members when one of their own is stricken with a chronic disease. Studies among patients undergoing dialysis reveal a positive relationship between social support and health care outcomes [34]. Including family members in the course of the disease to enable them to achieve empowerment may have an impact on the quality of care for patients in the pre-dialysis stage of kidney disease. The findings regarding the family members' responsibility concerning the patient's care and management of their medication are supported by previous research indicating that family members see themselves as important links between the patient and health-care professionals [4].

Listening to the family members' experiences and acknowledging their competence by focusing on their strength and encouraging them to tell their stories increased their sense of empowerment in health care [35,36]. Foster and colleagues claim that to cultivate an organization that includes and strengthens the whole family, there is a need to change health-care staff's strategies and attitudes towards the health-care system [37]. To focus on the whole family in health care is described as family-focused nursing [38] or the frontline in a clinical microsystem [39] and represents collaboration between patient, family and health-care staff for the purpose of providing high-quality care.

Empowerment in the present study had to do with family members' wanting to protect the patient from anxiety and being a positive force in changing lifestyle habits to improve the person's condition. However, this can be viewed as paternalism [40], in contrast to the creation of the trust and learning that are essential for empowerment of patients in outpatient care [23]. Thus, it is important to pay attention to empowerment of both the patient and family members to avoid paternalism or materialism. Family members' empowerment is an important component necessary but not in and of it self sufficient for achieving the larger outcome of quality patient care [41].

### Methodological Considerations

Every effort has been made to ensure trustworthiness of the findings by providing sufficient descriptions of empowerment grounded in family members' narrations. Hence, to make the results more credible, the quotations represent different family members and different relationships to the patient [42]. During the interview, the researcher summarized the participant's answer and asked whether the narrative had been correctly understood. The interviews in their entirety served as a point of reference throughout the analytical process when deeper understanding was needed of the meaning units, codes and sub-themes. In addition, the process of data collection was conducted, coded and analysed in Swedish. The quotations were translated directly from Swedish to English by an Englishman who spoke fluent Swedish so the original intended meaning would be preserved.

Procedures directed towards the credibility and transferability of the results were undertaken. Empowerment is a complex phenomenon and changes over time [43]. Interviewing family members on two occasions over time can increase the credibility of findings [22] as it can be seen as prolonged engagement. The time between the two interviews could have had an effect on the findings. The findings included more narratives about family members' experiences of responsibility for the patient in the second interview, in relation to the first interview. The analysis was divided into several stages of development, facilitating systematic inspection with an eye to the criterion of dependability. The findings show a high level of dependability without change over time [22].

The interviews on the second occasion were not conducted by the same researcher as on the first. This use of different researchers may have had a negative effect on the credibility of the findings. On the other hand, it could have had a *positive *effect if the same results emerged on the two occasions [22]. In the literature this is called investigator triangulation [19,44,45]. In addition, the data and research process were scrutinized by the research group to manage the bias that is embedded in close engagement [46]. For the sake of credibility, the analytical process has been illustrated so the reader may follow the researcher's interpretations. Choosing participants with a diversity of experiences also enhanced the credibility of the study [21].

Empowerment is not a term commonly used in society at large but is usually described in such terms as "power", "delegation of power to" and "self-determination". Researchers must have a pre-understanding of the concept. Therefore, a literature review on empowerment was performed before the data collection that guided the follow-up questions in the interview and should have increased the dependability of the findings [22]. This made it possible to validate the findings through appropriate formulation of the follow-up questions [47]. Furthermore, the interviews proceeded on the participant's own terms. That is, participants had the opportunity to share their experiences of health care in an open way without leading questions.

## Conclusions

To the best of our knowledge, this is the first qualitative study describing empowerment in outpatient care as experienced by the family of patients with pre-dialysis chronic kidney disease. The overall finding *Having strength to assume the responsibility *represents empowerment from the family members' perspective. The result elucidate experiences that are prerequisites for family members' empowerment in out-patient care, but also reveal experiences related to the absence of empowerment. Inviting family members to play an active role in care decisions and delivery (e.g. empowering them) may increase the quality of patient care. In addition, the findings stress the importance of including the family members' perspective in the education of health-care professionals so as to create a supportive environment that increases the family members' empowerment in outpatient care.

## Competing interests

The authors declare that they have no competing interests.

## Authors' contributions

AN and GA carried out the study design. AN and KW carried out the data collection. AN, GA, KW and DM participated in the data analysis and manuscript preparation. All authors read and approved the final manuscript.

## Pre-publication history

The pre-publication history for this paper can be accessed here:

http://www.biomedcentral.com/1472-6955/10/21/prepub
